# Text-mining of PubMed abstracts by natural language processing to create a public knowledge base on molecular mechanisms of bacterial enteropathogens

**DOI:** 10.1186/1471-2105-10-177

**Published:** 2009-06-10

**Authors:** Sam Zaremba, Mila Ramos-Santacruz, Thomas Hampton, Panna Shetty, Joel Fedorko, Jon Whitmore, John M Greene, Nicole T Perna, Jeremy D Glasner, Guy Plunkett, Matthew Shaker, David Pot

**Affiliations:** 1 ERIC-BRC, SRA International Inc, Global Health Sector, Rockville MD, 20852, USA; 2 14026 Marblestone Drive Clifton, VA 20124, USA; 3 Genome Center, University of Wisconsin, Madison WI, 53706, USA; 4 Laboratory of Genetics, University of Wisconsin, Madison WI, 53706, USA

## Abstract

**Background:**

The Enteropathogen Resource Integration Center (ERIC; ) has a goal of providing bioinformatics support for the scientific community researching enteropathogenic bacteria such as *Escherichia coli *and *Salmonella *spp. Rapid and accurate identification of experimental conclusions from the scientific literature is critical to support research in this field. Natural Language Processing (NLP), and in particular Information Extraction (IE) technology, can be a significant aid to this process.

**Description:**

We have trained a powerful, state-of-the-art IE technology on a corpus of abstracts from the microbial literature in PubMed to automatically identify and categorize biologically relevant entities and predicative relations. These relations include: Genes/Gene Products and their Roles; Gene Mutations and the resulting Phenotypes; and Organisms and their associated Pathogenicity. Evaluations on blind datasets show an F-measure average of greater than 90% for entities (genes, operons, etc.) and over 70% for relations (gene/gene product to role, etc). This IE capability, combined with text indexing and relational database technologies, constitute the core of our recently deployed text mining application.

**Conclusion:**

Our Text Mining application is available online on the ERIC website . The information retrieval interface displays a list of recently published enteropathogen literature abstracts, and also provides a search interface to execute custom queries by keyword, date range, etc. Upon selection, processed abstracts and the entities and relations extracted from them are retrieved from a relational database and marked up to highlight the entities and relations. The abstract also provides links from extracted genes and gene products to the ERIC Annotations database, thus providing access to comprehensive genomic annotations and adding value to both the text-mining and annotations systems.

## Background

With the advent of whole genome sequencing [1], decoding an organism's entire gene set and relating that information to its biology through annotation has become a central component of bioinformatics research. Annotation, the assignment of biological roles to portions of the genome sequence, is performed by many methods – from completely manual [2,3] to highly automated [4,5]. An important step in this process is establishing linkages between genome annotations and the experiments in the scientific literature that provide evidence supporting these annotations. Even inferences based on genome context are reliant on proximity to genes characterized in the literature. Once gene roles are established, they may then be used in automated pipelines to propagate annotations to orthologs in other genomes, a central premise of bioinformatics.

The Enteropathogen Resource Integration Center (ERIC, ) [6,7] is one of eight Bioinformatics Resource Centers (BRCs) for Biodefense and Emerging/Re-Emerging Infectious Diseases  funded by the National Institute of Allergy and Infectious Diseases (NIAID; ). ERIC serves as an information resource for enterobacteria from four genera on the NIAID list of select agents related to biodefense – *Escherichia*, *Shigella*, *Salmonella*, and *Yersinia*. Pathogens in these genera pose significant threats to human health directly or indirectly through crops and livestock, as evidenced by recent public health incidents linked to diarrheagenic *E. coli *and *Salmonella*. ERIC integrates data and bioinformatics tools to support genomic research on these microorganisms. At the heart of the system is ASAP, A Systematic Annotation Package for community analysis of genomes [8,9], providing its users with a database of high-quality annotations backed by evidence codes. This is achieved through the efforts of a dedicated team of annotators employing both manual examination of the experimental literature and automatic annotation methods.

The literature relevant to enterobacteria is extensive, particularly because this group includes the model organism *E. coli *K-12, and also because the family as a whole is experimentally tractable. Moreover, the accelerating pace of genome sequencing and the increasing number of genes addressed in single studies and high-throughput experimentation means that this corpus is growing. New approaches to extracting key findings and linking them to gene products are therefore urgently needed by database curators and the research community at large.

Here we describe a new tool for automated information extraction that we expect to improve the rate at which researchers and curators can survey new literature and identify experimental conclusions rapidly and accurately. We designed and built a text-mining application by using the NetOwl^® ^pattern-matching-based extraction engine and Oracle text indexing. The ERIC application was trained to extract entities and predicative relations relevant to molecular mechanisms of bacterial pathogenesis and functions of gene products. User-friendly interfaces help researchers and curators view the extraction results, create gene-centered lists of the relations, and link to the relevant ASAP annotations. By integrating text-mining and ASAP within the ERIC system, we provide added value to all systems and will hopefully drive community annotation and research efforts forward.

## Construction and content

### Development strategy

NetOwl^® ^Extractor  from SRA International, a state-of-the-art information extraction (IE) engine, was used to extract entities and relations of interest to enteropathogen research. NetOwl^®^, originally published as REES, is well-suited to the task because it has been designed as a scalable, portable system for entity, relation, and event extraction [10]. With over 12 years of research and development, NetOwl^® ^has a proven track record, and has demonstrated superior performance in various benchmarking events such as the Automated Content Extraction Evaluation sponsored by the National Institute of Standards and Technology [11].

NetOwl^® ^Extractor typically extracts relations between two entities. In the version implemented for ERIC, NetOwl^® ^extracts what we call "predicative relations", that is, relations where the first argument is an entity and the second argument is some text span, typically a phrase, denoting a complex concept such as function, phenotype, or pathogenicity. For the sake of simplicity, in the remainder of this communication we will use the terms "relations" to refer to "predicative relations".

The first step in development was to select concepts of interest for automated extraction. Since a central role of the ERIC-BRC is to understand mechanisms of pathogenesis through the sequencing and annotation of genomes, "genes" and the proteins encoded by them – the "gene products", were selected as concepts to extract. Enzymes, a particular class of gene products, are also relevant because their name frequently describes a gene role. Since it is important to associate entities and relations with the organism in which they are studied, "organism" and "strain" were also selected for extraction. The complete set of entities for extraction therefore consists of organism, strain, gene, operon (a functional grouping of multiple genes), gene product, and enzymes.

The second step was to choose the type of documents as input for the system. We chose to extract from abstracts because they are freely available through the PubMed service of the National Library of Medicine [12], whereas full texts of research studies are often only available by subscription. In addition, using abstracts rather than full text as input reduces computation time and enables higher throughput in the system. A corpus of abstracts was selected from two resources: literature already referenced in the ASAP genomic database as the source of annotations for its genomes; and periodic online searches of new publications from peer-reviewed domestic and international journals such as Journal of Bacteriology, Molecular Microbiology, and Journal of Biological Chemistry. A total of 465 abstracts were collected and randomly assigned to a training set (327 abstracts) or a blind set (138 abstracts). The PubMed IDs for each set are provided as TrainingSetPubMedIDs.txt and BlindSetPubMedIDs.txt as additional files 1 and 2, respectively. The training set is used for iterative development of the extraction system's lexicon and extraction rules. The blind set is used to measure how well the system performs on unseen data.

The third step was the formulation of specific mark-up guidelines for entities and for relations, which were developed by two molecular biologists in collaboration with a computational linguist. Using these guidelines, manual mark-up of the training and blind sets was performed by the biologists using a Graphics User Interface-based tool. Each abstract was marked up by one biologist and reviewed by the other. The guidelines were improved as more abstracts were acquired, and earlier mark-ups were revised accordingly.

#### Extraction software description

A set of extraction rules was developed based on the examples found in the training corpus. Extraction rules are generalizations of examples based on lexical (e.g. synonyms), syntactic (e.g. reordering), and domain knowledge. They are applied in an incremental and sequential fashion, whereby basic concepts (e.g. gene names) are identified first, and more complex concepts (e.g. relations) are identified by subsequent extraction rules.

In some cases, extraction rules exploit simple naming conventions for entities. For instance, enzymes often use the suffix "ase" (e.g. amylase). Bacterial gene names typically follow an accepted format [13] of three lower case letters followed by an upper case letter, all in italics (e.g. *hilA*). The corresponding gene product often has the same string of characters, only with the first letter capitalized and no italics (e.g. HilA). A similar pattern is often used to name operons: three lower case letters followed by more than one upper case letter representing the genes in the operon (e.g. *nikABCDE*). Our text mining application uses these capitalization conventions but does not rely on italicization since the input is plain text.

In other cases, extraction rules are context-sensitive. For instance, some names are ambiguous nouns (e.g. *fur*, *spa*) or insufficiently distinct (e.g. three lower case letters such as *rol*). Context-sensitive patterns allow for accurate extraction of those, maximizing recall (i.e. the number of true positives) while preserving precision (i.e. minimizing the number of false positives). In the interval from Jan 1 – Dec 1 2008, we found 150 PMID abstracts containing the exact term "fur". NetOwl^® ^extracts the term in only 25 of these and in 96/96 occurrences the context refers to a locus, gene or gene product. In the remaining 125 abstracts, fur is often used in another sense (e.g. fur seals, water or fur-assisted dispersal, etc.) but we have never seen these usages in an extraction. Similar terms such as "tag", "sac", etc. also yield high-precision extractions. We also tested performance in the partial absence of these disambiguation rules. Eleven contextual rules for 3-lower-case-letter names were removed and the extraction of operons in the blind set responded as follows: recall dropped by 4.4, and precision dropped by 2.5 (n = 310).

In another example of context-sensitivity, an extraction rule extracts a strain name preceded by a keyword like "serovar". It succeeds when it encounters a literal like "strain", "serovar" or "biovar" followed by an unknown word, defined as an item that does not belong to one of the built-in part of speech lists (e.g., noun, adverb). The match binding defines the expression that will be extracted as the strain name. Thus, the words "strain", "serovar" or "biovar" are used as contextual clues, but are not included in the extent of the extracted name.

Some extraction rules are list-based. For instance, since many of the organisms and strains relevant to the domain at hand are known, those are extracted through basic list lookup. Yet others are found dynamically; for instance, unknown names ending in "bacterium", as in *Agrobacterium *are extracted as organism names. Some unknown strain names are also identified in context, for instance when they are coordinated with known strain names as "Nepal516" in the context "strains Antiqua and Nepal516".

Relation extraction is a far more complex task. It requires the entities involved to be correctly identified, and it must allow for far more variability in the way concepts are expressed. To extract relations, the task is further complicated by the fact that one of the two arguments involved is a text span, often a phrase, denoting a complex concept such as a function, mutation result, or pathogenesis. Phrases are typically far more complex syntactically and semantically than named entities.

The predicative relations currently extracted for ERIC are of three types:

1. a gene (or operon or gene_product) and its role, e.g. "phage-shock-protein A (*pspA*) operon encodes an extracytoplasmic stress response system"

2. a mutant form of a gene and its phenotype, e.g. "The *xdhA *mutant grew faster with aspartate as a nitrogen source"

3. an organism (or strain) and its pathogenesis, e.g. "*Yersinia enterocolitica*, an important food- and water-borne enteric pathogen"

Our general approach is to identify phrases that may denote a function, phenotype, or pathogenesis and then establish a link with an entity. For instance, in the phrase "*sitB *encodes an ATP-binding protein", the system first identifies the expression "encodes an ATP-binding protein" as a function phrase based on an extraction rule that looks for an encode-like predicate and a protein-like element. A later extraction rule creates a link between the gene entity *sitB *and the function phrase. The results can be used immediately to help infer a function for a gene as described in the literature.

Table 1 lists the number of extraction rules employed by the ERIC NetOwl^® ^extractor. Additional file 3 (paraphrased_description_of_rules.doc) provides more detail on rules used in the application.

**Table 1:** Number of extraction rules implemented in ERIC NetOwl^®^

**Entities**	**Number of Extraction Rules**	**Relations**	**Number of Extraction Rules**
Organism	5	Gene or Gene Product Roles	150
Strain	18	Mutation Phenotypes	42
Enzyme	5	Organism Pathogenesis	9
Gene	18		
Gene Product	20		
Operon	31		

As evidenced by this table, some concepts require many more extraction rules than others. In the case of gene roles, the large number of extraction rules correlates with the larger number of expressions that may denote a role. The more variability in the way a concept is expressed and/or the more ambiguous an expression is, the larger the number of extraction rules needed to capture those expressions accurately.

#### Evaluation

We used an automated scoring tool to assess the effectiveness of the extraction system on both the training set (327 abstracts) and blind set (138 abstracts). The tool measures accuracy using standard IE metrics: Recall (R), Precision (P), and F-measure. Recall is the percentage of the system's correct hits or "true positives" compared to all human-annotated items, including those that were missed or "false negatives" (TP/(TP+FN)). Precision is the percentage of true positives among all the extracted items, including spurious hits or "false positives" (TP/(TP+FP)). The F-Measure is a weighted average, defined as (2 × recall × precision)/(recall + precision). The closer the precision and recall scores are, the closer the F-Measure score will be to a standard average; if the recall and precision scores are far apart, the F-Measure will drop substantially. The final recall, precision, and F-measure scores on the blind set for entity and relation extraction are presented in Table 2.

**Table 2:** Performance scores on entities and relations in Blind set (138 abstracts)

**Entities**	**Recall**	**Precision**	**F Measure**	**Relations**	**Recall**	**Precision**	**F Measure**
Organism (1362 entities)	92.0	98.1	94.9	Gene or Gene Product Roles (615 relations)	62.5	82.9	70.9
Strain (554 entities)	81.1	82.9	81.9	Mutation Phenotypes (149 relations)	58.5	77.8	66.7
Enzyme (386 entities)	85.7	81.6	83.5	Organism Pathogenesis (34 relations)	68.9	83.1	75.1
Gene (916 entities)	93.6	93.7	93.6				
Gene Product (1425 entities)	92.3	94.8	93.5				
Operon (310 entities)	96.2	93.0	94.5				

We found no significant dependence on system performance as a function of journal source. The blind set abstracts were subdivided by category: specialty microbial journals (e.g. Journal of Bacteriology, Microbiology, etc.) vs. general biological journals (Journal of Biological Chemistry, Proc. Nat. Acad. Sci. USA, etc.) and re-tested. For the gene-role, mutation-phenotype, and organism-pathogenesis relationships, the F-measures were respectively 70.4 vs. 69.2, 63.4 vs. 69.8, and 82.5 vs. 73.3. (The relatively large difference in the organism-pathogenesis comparison may not be statistically significant due to the small sample size.)

#### Database

**Figure 1:**
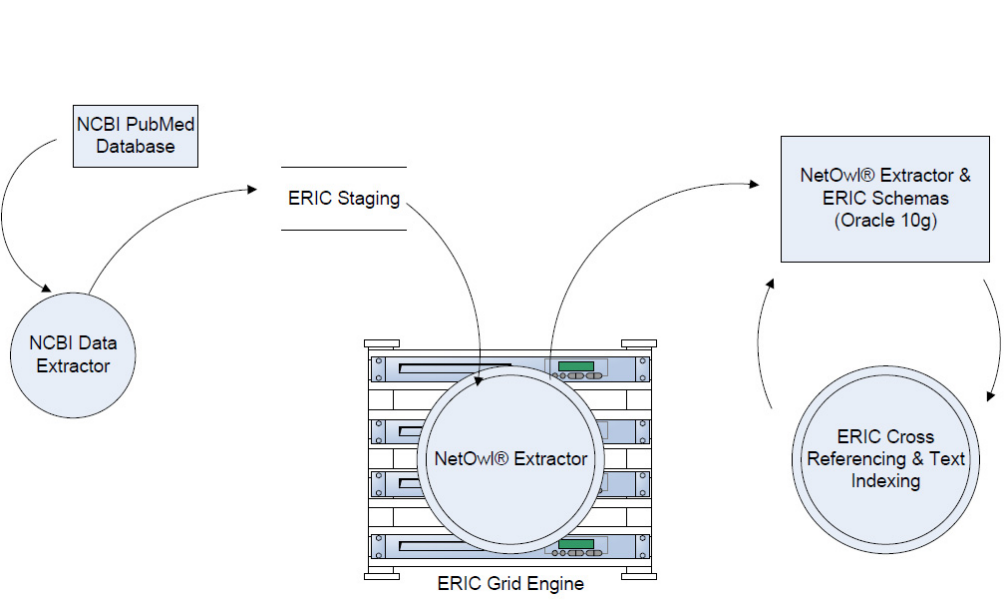
**An overview of the ERIC Literature Text Mining population process**.

PubMed abstracts are obtained daily via the ERIC NCBI Data Extractor which utilizes the ESearch and EFetch NCBI Entrez Programming Utilities [14]. In addition, archived abstracts are being collected to extend historic coverage of the literature. Abstracts are processed through the NetOwl^® ^Extractor, a multi-threaded application which utilizes the Sun N1 Grid Engine, the software hosting ERIC's distributed computing environment. This distributed computing environment coordinates between multiple servers to appear to an application as one large computational resource, so NetOwl^® ^Extractor can ingest documents more quickly and efficiently. Under these conditions, the average processing time (extraction plus ingestion) for documents, considering both contemporary and historical abstracts, is 120 Megabytes/hour. In a typical month, this translates to an abstract processed every 1.49 seconds of operation. The extraction results are stored in an Oracle 10 g relational database, cross-referenced to ERIC genomes, and text indexed for search and retrieval. This process is illustrated in Figure 1 below. As of February 2009, over 6 million PubMed abstracts from June 1999 forward are available for searching and viewing.

#### Utility

**Figure 2:**
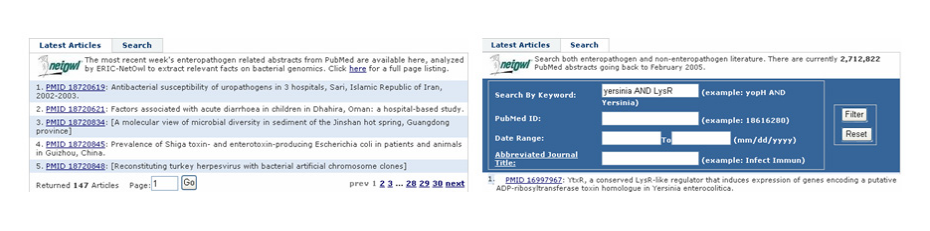
**(Left) The Latest Articles tab lists PubMed abstracts involving enteropathogens published over the previous 7 days**. (Right) The Search tab supports query by keyword(s) and phrases, PMID, date range, and/or journal. The PMID link of a title retrieves the abstract in the ERIC text mining interface.

Our text mining application is freely available for use online at the ERIC website . The user interface is currently a two-tabbed portlet for retrieving PubMed abstracts processed by the NetOwl^® ^IE engine (Figure 2). The 'Latest Articles' tab lists new enteropathogen literature published over the preceding week. Alternatively, the 'Search' tab allows users to query the database for articles by combining specific keywords, journals, and/or date ranges. The Search function allows for searching PubMed abstracts beyond the enteropathogen literature. As a caveat, the ERIC instance of NetOwl^® ^was trained on the conventions and syntax of microbial literature. In other biological domains that do not adhere to those conventions, the evaluation metrics may not attain the levels described above.

**Figure 3:**
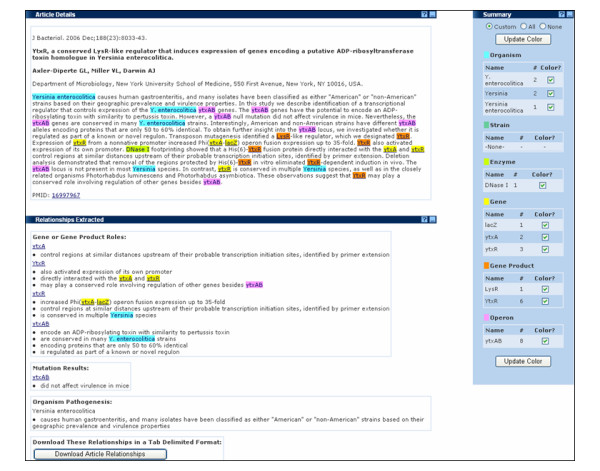
**ERIC text mining interface of a PubMed abstract processed by NetOwl^®^**.

On either tab, clicking on a PMID link retrieves the abstract. An abstract is displayed in a three panel interface (Figure 3). At the top, the Article Details panel displays the full text of the abstract. Mentions of organisms (e.g. *Y. enterocolitica*), strains, enzymes (e.g. DNase I), genes (e.g. *ytxA*), gene products (e.g. LysR), and operons (e.g. *ytxAB*) are highlighted and color-coded by type. A PubMed link following the text points to the full entry at NCBI.

A Summary panel to the right of the interface tallies the number of occurrences of entities and allows the user to suppress highlighting of any item in the Abstract Details as desired.

**Figure 4:**
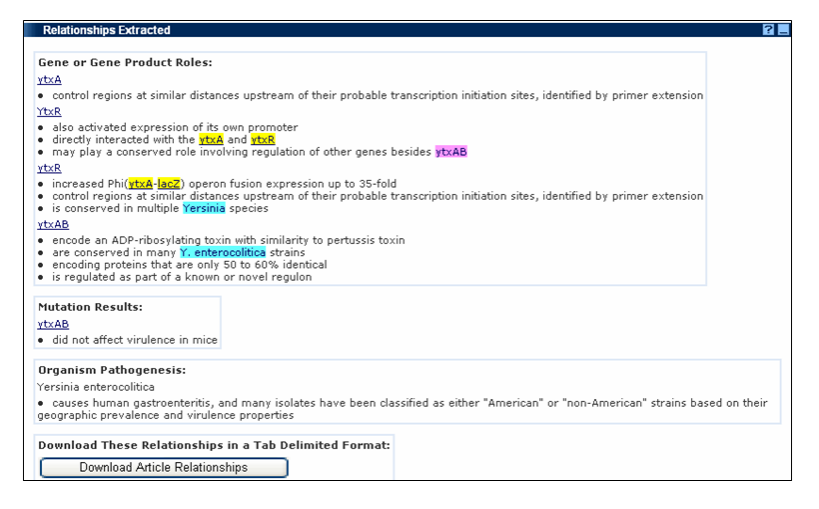
**Detail of the Relationships Extracted panel on the ERIC text mining interface**.

The Relationships Extracted panel at the bottom (detailed in Figure 4) summarizes the automatically extracted predicative relations that fit into one of the three categories:

• Gene or Gene Product Roles, e.g. YtxR – also activated expression of its own promoter

• Mutation Results, e.g. *ytxAB *– did not affect virulence in mice

• Organism Pathogenesis, e.g. *Yersinia enterocolitica *– causes human gastroenteritis, and many isolates have been classified as either "American" or "non-American" strains based on their geographic prevalence and virulence properties

Within each category, all relations associated with a single entity are grouped together, quickly allowing the user to see the key findings that are reported in the abstract. The accuracy of any extracted phrase can be validated by referring to the highlighted entity in the Article Details pane for its original context. For a permanent record of the relations, the Download Article Relationships button on this panel will generate a tab-delimited file of all the extracted relations, which can be used programmatically, or opened in a text editor or a spreadsheet.

**Figure 5:**
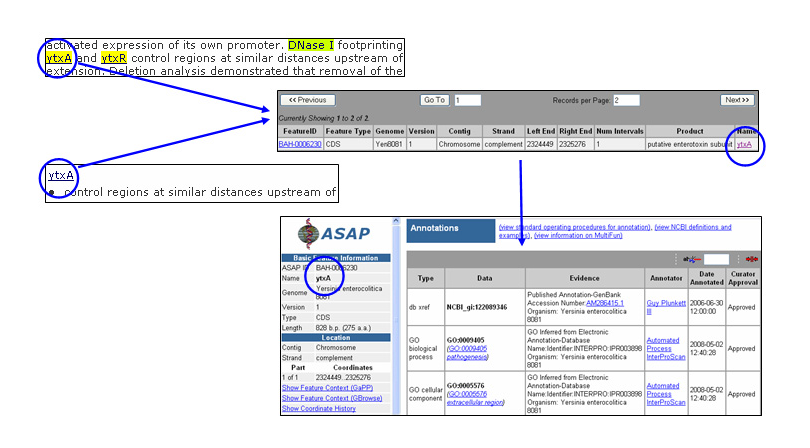
**Montage shows workflow from an extracted gene/gene products in the text-mining interface, to the ASAP annotations database**.

Clicking a highlighted gene or gene product in either the Article Details or Relationships Extracted panel initiates a search of the ASAP database. The result is a table with all genes by that name contained in the genomes housed in ERIC (Figure 5). The table may include the gene in the specific genome mentioned in the abstract, the most experimentally studied genome, and/or the genome of most interest to the individual user. Clicking on an individual entry opens the respective ASAP Detailed Feature page, which provides other relevant functional annotations and supports community annotation efforts (see Discussion below).

## Discussion

Several text-mining applications on the Web have been described in the literature in recent years, [15-17], often focusing on specialty areas in biomedical research. For a recent review, see Krallinger, et al. [18]. Within the microbial domain, NLP was evaluated for mining the *E. coli *K-12 literature to assist in curation of the RegulonDB database of regulatory networks [19], and the widely-used Textpresso search engine [20] is now available to search the RegulonDB database itself [21].

ERIC text mining is directed towards the research literature concerning enteropathogen molecular biology and pathogenesis. A state-of-the-art IE engine was trained on a corpus of relevant abstracts and integrated with a text-indexing relational database and intuitive retrieval and display interfaces. Operationally, the system presents several potential benefits to researchers active in the field.

The current system is highly accurate on the named entity recognition task. Even though bacterial gene naming follows well-established conventions, gene names are often ambiguous words (e.g., fur, spa). Context-sensitive rules allowed us to handle those with high accuracy.

Many applications extract complete sentences meeting programmatic criteria. Natural language presents complex syntactical structures, and many sentences contain several distinct entities and their relations. The annotator must expend considerable effort in analyzing sentences and clauses for the meaningful information. Parsing such sentences into a format for upload into a database requires text editing or programming expertise.

Our present text-mining application assumes some of this effort by extracting phrases from within sentences and organizing gene-centered lists of each separate entity and its predicative relations. The results are more concise for the reader and more amenable to database applications. For example, in the sentence "Two unrelated enzymes with R5P isomerase activity were first identified in *Escherichia coli*, RpiA and RpiB" [22], the role "...enzymes with R5P isomerase activity, etc." was automatically assigned to two *separate *entities: RpiB and RpiA. The ERIC text mining approach also demonstrates its versatility by extracting multiple, independent ideas from a single sentence. In the sentence "It is known that the expression of *iscS *can be negatively regulated by IscR, the first gene product of *iscRSUA*-*hscBA*-*fdx*." [23], the application extracts two separate gene-role relations:

1. ***iscS ***– can be negatively regulated by IscR

2. ***IscR ***– the first gene product of *iscRSUA*-*hscBA*-*fdx*

The present format has the potential of being more "database-ready" than complete sentences, since it may require little or in some cases no further manipulation in a text editor.

**Figure 6:**
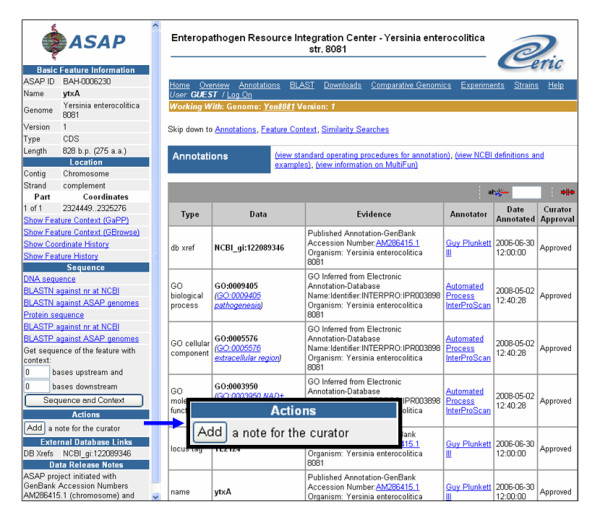
**Detailed Feature page in ERIC-ASAP**. Community users viewing newly extracted information may alert ERIC via the Add a note to the curator button (inset).

The integration of the text mining interface to the ERIC-ASAP Annotations database benefits the scientific researcher and enhances the value of both systems. For a researcher viewing an extracted gene role or mutation phenotype in the text mining interface, the annotations on an ASAP Detailed Feature page can give context to the new experimental information and stimulate hypotheses. For those researchers wishing to contribute to community annotation, anyone can leave a note alerting the ERIC staff to a significant new piece of information. The Add a note to the curator button (Figure 6) opens an intuitive notepad for anonymous communication with ERIC regarding a specific gene.

Finally, users who choose to register as ERIC account holders (via ) can request Annotator or Curator status on any genome, and thereby assume greater control over addition of new information. ERIC includes tools that assist curators with propagation of annotations to related features in other genomes so that potentially relevant functional information about genes is available to researchers working on different but related enteropathogen genomes.

The current implementation of this IE-based tool by ERIC-BRC effectively highlights relevant facts extracted from scientific abstracts. We anticipate developing further interfaces to the extracted information, including gene by gene summaries across many abstracts, and provision of web services to make the data more readily available for other users computationally. A text download of extracted data from articles citing *Escherichia*, *Salmonella*, *Shigella *and *Yersinia *has been made available for further bioinformatics analysis outside the system. Moreover, the extraction system itself could be enhanced by extending the current extraction rules to extract other relevant predicative relations such as subcellular location and similarity.

Expert curation, such as that provided by ERIC's staff, remains necessary before the results of NLP analyses can be directly incorporated into curated genome annotation databases. Since no system is completely accurate, it is essential to have a scientist review the extracted data before deposition into a database. One challenge is overcoming inter-species ambiguity in extraction and curation. As far as possible, one must avoid taking annotations that may be correct in one genome (e.g. known or putative virulence factor) and attaching them to orthologs in another genome in which they are unproven or perhaps even incorrect. The ERIC environment may provide assistance in this regard. Since NetOwl^® ^extraction of organism and strain details from the abstracts already shows good F measure scores, perhaps this information can be used to more accurately direct users or future versions of the application to the respective feature in the correct genome.

## Conclusion

A natural language processing database and application was developed and recently launched online at the ERIC website . The application automatically processes biomedical abstracts on a daily basis, and extracts entities and relations relevant to molecular mechanisms of bacteria, including pathogenesis. Results of the extractions are searchable and are displayed in interfaces that allow users to rapidly identify the conclusions presented in the abstracts, and create summaries of the genomic relations described in them. Seamless integration of the system with ASAP, a curated community-based annotations database, with access to additional sequence analysis tools in the ERIC portal, provides greater context to these conclusions and enhances the ability of researchers to generate working hypotheses.

## Availability and requirements

The ERIC text mining application  is freely accessible for use. The ERIC homepage is: . The application supports popular browsers on Windows and Mac OS X. There are no restrictions to use by non-academics.

## Authors' contributions

SZ and DP collected and annotated abstracts, assisted MRS on the mark-up guidelines, analyzed interim results to enhance extraction rules, and advised on workflow and interface design. MRS formalized and implemented extraction rules, developed the extraction component of the ERIC Text Mining application, and ran the performance evaluations. PS, TH and JW designed and implemented interfaces and workflows. JF implemented and manages the ERIC Text Mining database. JMG, NTP, JDG, GP, and MS provided valuable suggestions on utility and design. SZ drafted the manuscript with assistance from DP, MRS, JDG, NTP, and JMG. MS is the ERIC Team Project Manager. All authors read and approved the final manuscript.

## Supplementary Material

Additional file 1 **Training Set PubMed IDs**. Tab-delimited text file with list of PubMed IDs used in training set. Click here for file

Additional file 2 **Blind Set PubMed IDs**. Tab-delimited text file with list of PubMed IDs used in blind set. Click here for file

Additional file 3 **Paraphrased description of rules**. Word document with paraphrased description of extraction rules. Click here for file
