# Supplementary material for: Text-mining of PubMed abstracts by natural language processing to create a public knowledge base on molecular mechanisms of bacterial enteropathogens
Source: BMC Bioinformatics. 2009 Jun 10;10:177. doi: 10.1186/1471-2105-10-177 (PMC2704210; doi:10.1186/1471-2105-10-177)
Supplement: Additional file 3 — Paraphrased description of rules. Word document with paraphrased description of extraction rules. [file 1471-2105-10-177-S3.doc]

In the ERIC system, we developed two types of extraction rules to identify named entities. There are rules based on a name’s internal structure, prefixes, or suffixes. For instance:

1. ullu

This rule identifies gene product names that consist of a four-letter string of the form uppercase-lowercase-lowercase-uppercase, e.g. HilA.

1. lllu

This rule identifies gene names that consist of a four-letter string of the form lowercase-lowercase-lowercase-uppercase, e.g. hilA.

1. llluu+ (variable number of u’s)

An operon entity is defined by a multi-letter string of the form lowercase-lowercase-lowercase followed by two or more uppercase letters, e.g. iucABCD.

4. organism_ending_in_bacterium

This rule identifies organism names that consist of a word ending with such as bacterium, bacillus, coccus, or cocci, e.g. *Lactobacillus acidophilus*.

5. string_ending_in_ase

This rule identifies enzyme names that consist of a word ending in “ase(s)”, e.g. GTPase. Optional elements of this rule allowing for premodifiers include:

- - the word “type” followed by a Roman numeral, e.g. type II topoisomerases.
  - a word, followed by a hyphen, followed by the word “dependent” or “sensitive”, e.g. Na+-dependent PPases, vanadate-sensitive ATPase.

There are also entity-naming rules based on a context. For instance:

6. ull_dash_dependent

This rule identifies gene product names that consist of a three-letter string of the form uppercase-lowercase-lowercase when they are followed by a hyphen and the word “dependent”, e.g. Src-dependent actin polymerization.

7. gene_product_comma_an_enzyme

This rule identifies gene product names that consist of a three-or-four-letter string of the form uppercase-lowercase-lowercase or uppercase-lowercase-lowercase-uppercase when followed by a comma, a determiner (e.g. an, one), and an enzyme name identified previously, e.g. Frp, a flavin oxidoreductase from *Vibrio harveyi*.

8. string_apposed_to_the_word_strain

A strain entity is defined by a mixed-case multi-character string when it is apposed to the keyword <strain>, e.g. The opgGH mutation also resulted in a 2 log increase in the LD(50) in mice compared to the wild-type strain SL1344.

Extraction rules are applied in a sequential manner, whereby simpler and high-precision rules are applied first, and more complex rules are applied later and may rely on items identified by previous rules. For example, an early rule may identify an enzyme name and a later rule may identify an alternative, abbreviated form of the same enzyme in context.

Extraction rules for identifying relationships can also be considered as two categories. There are rules based on appositions. For instance:

9. the_mod_regulator_comma_entity

This rule identifies a link between a gene, gene product, or operon and a pre-appositive with the basic structure determiner+modifier+regulator, e.g. the arginine repressor argR.

10. entity_a_cause_of_disease

This rule creates an organism-pathogenesis link between an organism or strain and an apposition with the basic syntactic structure determiner+cause+of+disease, e.g. *Shigella*, the causative agent of bacillary dysentery.

And there are rules based on verb phrases. For instance:

11. mutation_of_gene_causes

This rule identifies a mutation-phenotype link between a gene, gene product, or operon mutant and a verb phrase that denotes affecting (e.g. cause, alter, activate, etc.), e.g. the fliA mutant caused wild-type lethality.

12. entity_belongs_to_family_of_regulators

This rule creates a gene-role link between a gene or gene product and a verb phrase with the basic syntactic structure: belongs+to+family/subfamily+of+regulator, e.g. SitC lipoprotein, which belongs to a family of ATP binding cluster (ABC) transporter substrate-binding proteins.
